# Undergraduate Internal Medicine Training and Medical University Curriculum in Romania

**DOI:** 10.4274/balkanmedj.2018.1289

**Published:** 2019-01-01

**Authors:** Polliana Mihaela Leru, Vlad Florin Anton

**Affiliations:** 1 Carol Davila University of Medicine and Pharmacy, Bucharest, Romania; 2 Colentina Clinical Hospital, Bucharest, Romania

To the Editor,

We refer to an interesting paper published in your journal in 2014 on the important topic of undergraduate medical curriculum in Turkey, evaluated by residents (1), and we intend to add students’ opinion on some aspects of university medical education in our country, focusing on internal medicine. Internal medicine is one of the oldest medical specialties that can be considered as the cornerstone of the health care system in Western societies (2). Recent research has demonstrated the importance of internal medicine and the need to harmonize training in European countries, due to the increasing complexity of patient care and the migration of physicians. The situation of internal medicine university teaching is different from one country to another, possibly leading to difficulties in practicing medical profession (3). In our country, the university Internal medicine teaching was replaced by subspecialties by the end of 1990s, and the consequences of this change have not yet been evaluated. The aim of our study was to assess the current opinion of medical students about internal medicine training and their general educational needs and expectations to draw some suggestions for the improvement of medical university curriculum.

A questionnaire consisting of 25 questions, posted online on social network groups during 6 months, was addressed to the last three study years’ students from the largest medical university. The questionnaire was completed and processed using eSurv platform. We obtained 798 completed questionnaires, representing a response rate of 27.96%. The questions were grouped into the following four major topics: general opinion about medical training during university studies, opinion about internal medicine training, educational needs, and suggestions for improvement of university teaching (Table 1).

Internal medicine was considered as a still actual and useful specialty by 76.8% of responders. Comparing with related specialties, 71.8% of the responders considered that medical semiology cannot replace internal medicine in medical teaching and 67.3% of the students considered that family medicine cannot replace internal medicine in ambulatory care. More than 70% of responders showed a general dissatisfaction with clinical medical university education, 39.9% considered that practical training should be improved, while 58.0% of them considered that both practical and theoretical training needs improvement. Regarding medical university curriculum, 64.2% answered that this should be revised and completed with new topics. Another 68.5% of responders considered that internal medicine should be part of the standard medical university curriculum and 54% recognized their need for longer practical training. The majority of students considered that internal medicine teaching should be improved and updated, avoiding redundant lectures, according to the development of derived subspecialties. The primary limitation of our study is the low response rate, despite almost 800 responders. Continuation of the study is recommended, targeting a higher response rate.

Despite not being based on a validated questionnaire and a possible research bias, our study revealed that a significant proportion of medical students are not satisfied with their medical education during university studies and request better practical training. Students consider that Internal medicine training has an important place in medical university curriculum, which should be improved and revised according to university education priorities and international standards.

## Figures and Tables

**Table 1:**
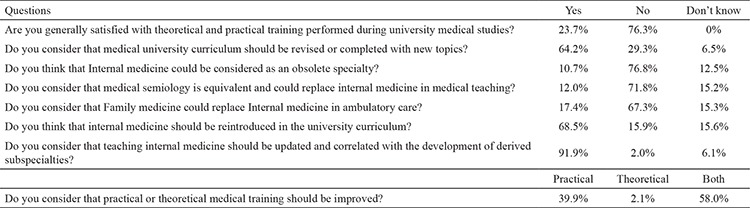
Relevant questions and students’ answers

## References

[1] Budakoğlu Iİ, Coşkun O, Ergün MA (2014). National undergraduate medical core curriculum in Turkey: evaluation of residents. Balkan Med J.

[2] Bauer W, Schumm-Draeger PM, Koebberling J, Gjoerup T, Garcia Alegria JJ, Ferreira F, et al (2005). The EFIM Working Group on Political Issues in Internal Medicine in Europe, Political issues in internal medicine in Europe. A position paper. Eur J Intern Med.

[3] Cranston M, Semple C, Duckitt R, Vardi M, Lindgren S, Davidson C, et al (2013). European Board of Internal Medicine Competencies Working Group. The practice of internal medicine in Europe: organisation, clinical conditions and procedures. Eur J Intern Med.

